# Potent anti-coronaviral activity of pateamines and new insights into their mode of action

**DOI:** 10.1016/j.heliyon.2024.e33409

**Published:** 2024-06-22

**Authors:** Francesca Magari, Henri Messner, Florian Salisch, Stella M. Schmelzle, Ger van Zandbergen, Alois Fürstner, John Ziebuhr, Andreas Heine, Christin Müller-Ruttloff, Arnold Grünweller

**Affiliations:** a Institute of Pharmaceutical Chemistry, Philipps University Marburg, 35032, Marburg, Germany; b Institute of Medical Virology, Justus Liebig University Giessen, 35392, Giessen, Germany; c Division of Immunology, Paul-Ehrlich-Institute, 63225, Langen, Germany; d Institute for Immunology, University Medical Center of the Johannes Gutenberg University of Mainz, Mainz, Germany; e Research Center for Immunotherapy (FZI), University Medical Center, Johannes Gutenberg-University Mainz, Mainz, Germany; f Max-Planck-Institut für Kohlenforschung, 45470, Mülheim Ruhr, Germany

**Keywords:** Pateamines, Rocaglates, eIF4A, Antiviral activity, Coronavirus, RNA clamping

## Abstract

Pateamines, derived from the sponge *Mycale hentscheli*, function as inhibitors of the RNA helicase eIF4A and exhibit promising antiviral and anticancer properties. eIF4A plays a pivotal role in unwinding stable RNA structures within the 5′-UTR of selected mRNAs, facilitating the binding of the 43S preinitiation complex during translation initiation. Pateamines function by clamping RNA substrates onto the eIF4A surface, effectively preventing eIF4A from carrying out the unwinding step. Rocaglates, a compound class isolated from plants of the genus *Aglaia*, target the same binding pocket on eIF4A, and based on structural data, a similar mode of action has been proposed for pateamines and rocaglates. In this study, we conducted a detailed characterization of pateamines' binding mode and assessed their antiviral activity against human pathogenic coronaviruses (human coronavirus 229E (HCoV-229E), Severe acute respiratory syndrome coronavirus 2 (SARS-CoV-2)). Our findings reveal significant differences in the binding behavior of pateamines compared to rocaglates when interacting with an eIF4A-RNA complex. We also observed that pateamines do not depend on the presence of a polypurine tract in the RNA substrate for efficient RNA clamping, as it is the case for rocaglates. Most notably, pateamines demonstrate potent antiviral activity against coronaviruses in the low nanomolar range. Consequently, pateamines broaden our toolbox for combating viruses that rely on the host enzyme eIF4A to conduct their viral protein synthesis, indicating a possible future treatment strategy against new or re-emerging pathogenic viruses.

## Highlights

• Pateamines are marine macrodiolides with potent anti-coronaviral activity.
• Pateamines inhibit viral protein synthesis in coronavirus infected cells by blocking the RNA helicase eIF4A.
• Clamping of RNA by pateamines on the surface of eIF4A is independent from stacking interactions with amino acids.
• Pateamines do not require a polypurine tract for clamping RNA substrates on eIF4A.
• Guanines embedded in a sequence of the type (NG)_x_ are crucial for RNA clamping by pateamines.

## Graphical abstract

**Figure:**
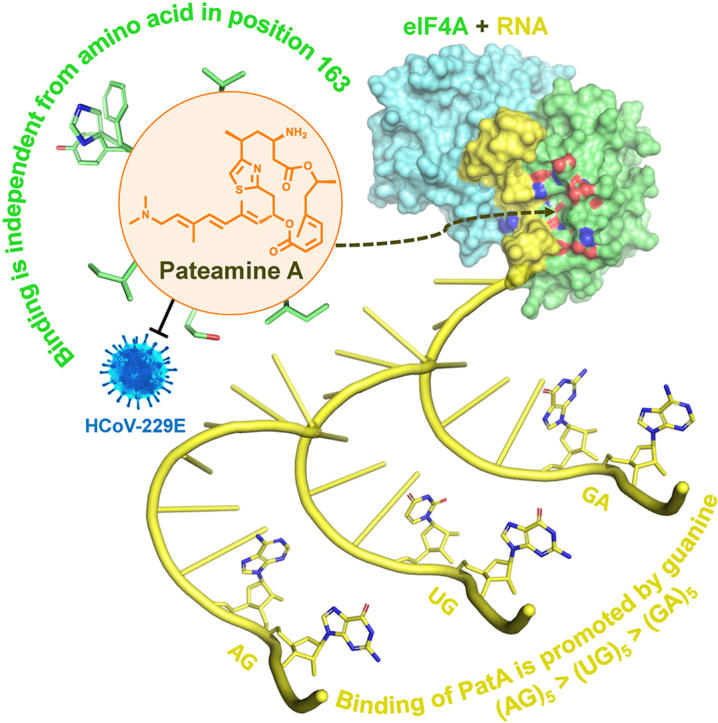

## 1 Introduction

Pateamine A (PatA) is a marine macrodiolide isolated from the sponge *Mycale hentscheli*, known for its anticancer and antiviral properties by blocking translation initiation in eukaryotic cells [[1], [2], [3]]. Translation initiation is a complex process involving multiple factors, including the RNA helicase eIF4A, an essential component of the heterotrimeric eIF4F complex that binds to the cap-structure of mRNAs via eIF4E [4]. During this process, eIF4A unwinds stable RNA structures in 5′-untranslated regions (5′-UTRs), enabling binding of the 43S preinitiation complex and ribosome loading [5,6].

Due to its vital role in cellular growth and proliferation, eIF4A inhibitors have been explored as potential anticancer agents *in vitro, in vivo*, and in clinical trials [[7], [8], [9], [10]]. Recent studies have revealed the involvement of eIF4A in the replication of several virus families, including filo-, picorna-, flavi-, and coronaviruses [4]. A large number of different viruses use eIF4A to facilitate the translation of their own viral mRNAs, thereby promoting replication [4]. For example, the specific eIF4A inhibitor silvestrol, a natural rocaglate, has demonstrated potent antiviral activity against Ebola-, Lassa-, Hepatitis E−, Zika-, Chikungunya- and Coronaviruses [[11], [12], [13], [14], [15]]. In addition, other eIF4A inhibitors, such as hippuristanol or PatA, displayed antiviral activity e.g. against Sindbis-, Zika- and Influenza A viruses [2,3,16], indicating the importance of eIF4A as a broad-spectrum antiviral target. Consequently, eIF4A inhibitors hold therapeutic potential in combating cancer, viral infections as well as infections with different pathogens, like *Plasmodium falciparum*, *Candida auris* or *Toxoplasma gondii* [[17], [18], [19]]. However, further evaluation is needed to fully understand the potentially different modes of action of various eIF4A inhibitors.

For rocaglates, it has been shown that they function as RNA clamps in a polypurine-dependent manner [20]. Co-crystallization studies involving RocA or desmethyl-PatA, a structurally simplified synthetic analogue of PatA [21], with eIF4A and a (AG)_5_ polypurine RNA have revealed that, despite the very different structures of both compounds, they can bind to the same pocket on the eIF4A surface and interact with two purines through π-π stacking interactions [22,23]. This suggests a remarkable case of molecular mimicry occurring in two vastly different organisms. Nevertheless, targeting protein-RNA interfaces is still an underestimated strategy in drug development.

In this study, we analyzed and compared two different pateamines (PatA and desmethyl-desamino-PatA (DMDAPatA), with different rocaglates regarding their mode of action and antiviral activity. First, we used thermal shift assays (TSA) to investigate the binding of pateamines to a wide range of eIF4A variants and RNA substrates. Our findings suggest that pateamines have a less restrictive binding mode to the eIF4A-RNA complex in terms of the interaction with defined amino acids compared to rocaglates. This reduced amino acid restriction could be confirmed in rocaglate-resistant *Leishmania*, where PatA serves as an effective eIF4A inhibitor. In contrast to rocaglates, pateamines do not rely on a polypurine tract for clamping the RNA substrate. Furthermore, we discovered a potent antiviral activity of pateamines against the human coronaviruses 229E (HCoV-229E) and Middle East respiratory syndrome-CoV (MERS-CoV) comparable to those of rocaglates. Therefore, pateamines display a suitable alternative for rocaglates with (i) similar antiviral efficacy, but (ii) less restrictive substrate specificity enabling eIF4A inhibition in rocaglate-resistant organisms. In summary, our results underscore the broad therapeutic potential of eIF4A inhibitors in combating infections with viruses and pathogens.

## 2 Material and methods

### 2.1 Cell culture

Human fetal lung fibroblasts (MRC-5 cells, ATCC, CCL-171) and Huh-7 cells (JCRB cell bank, JCRB0403) were grown in Dulbecco's modified Eagle's medium (DMEM) supplemented with 10 % fetal calf serum (FCS), 100 U/ml penicillin and 100 μg/ml streptomycin at 37 °C in an atmosphere containing 5 % CO_2_. Human hepatocyte carcinoma cells (HepG2 cells, ATCC HB-8065) were grown in Iscove's Dulbecco Medium (IMDM) supplemented with 10 % FCS and 1 % of Penicillin/Streptomycin at 37 °C in an atmosphere containing 5 % CO_2_.

### 2.2 Compounds

PatA and DMDAPatA (purity >95 %) were prepared in the laboratory of Prof. A. Fürstner from the Max-Planck-Institut für Kohlenforschung (Mülheim and Ruhr, Germany) [21] and provided by Prof. Fürstner. Silvestrol was obtained from the Sarawak Biodiversity Centre (Kuching; North-Borneo, Malaysia; purity >99 %). A 6 mM stock solution was prepared in DMSO and diluted in DMEM. Control cells were treated with corresponding DMSO dilutions lacking Silvestrol. CR-31-B (−) [24] was dissolved in DMSO at a concentration of 10 mM and stored at −20 °C.

### 2.3 Thermal shift assay

TSA experiments were performed on a real-time PCR system (QuantStudio™ 3, Applied Biosystems, Waltham, MA, USA) in a MicroAmp™ Fast Optical 96-well plate (Applied Biosystems, Waltham, MA, USA) using QuantStudio™ Design & Analysis software (version 1.4.2.). 5 μM of recombinant *human* eIF4A1_(19-406)_ was incubated with 50 μM of a polypurine RNA (AG)_5_ (Biomers, Ulm, Germany), 1 mM AMP-PNP (Roche, Basel, Switzerland), 100 μM of inhibitors (silvestrol, PatA, DMDAPatA) and 75 μM of SYPRO Orange (S6650, Invitrogen, Carlsbad, CA, USA) in 20 mM HEPES–KOH buffer pH 7.5, 100 mM KCl, 5 mM MgCl_2_, 1 mM DTT and 10 % (v/v) glycerol at RT. In the first step, the protein sample was subjected to a heating rate of 1.6 °C/s until a temperature of 10 °C was reached and kept constant for 2 min. In the second step, the temperature was increased by 0.05 °C/s until a temperature of 95 °C was reached and kept constant for 1 min. In the final step, the temperature was decreased by 1.6 °C/s until 10 °C and kept constant for 1 min. The wavelength of the fluorescence scan for excitation and emission was set to the spectroscopic maxima of SYPRO® Orange (472 nm and 570 nm, respectively). The melting curves were analyzed using Protein Thermal Shift Software (version 1.3) from Thermo Fisher Scientific.

### 2.4 Cell toxicity

Cell viability of MRC-5 cells in the presence of either PatA or DMDAPatA was determined by using the 3-(4.5-dimethylthiazol-2-yl)-2.5-diphenyl-2*H*-tetrazoliumbromide (MTT) assay. The cytotoxic concentration 50 % (CC_50_) of either PatA or DMDAPatA was determined by incubating cells, which were seeded near confluency in DMEM medium, with serial dilutions of each compound in a 96-well format. After incubation for 24 h, 100 μL of MTT-mix (DMEM supplemented with 10 % FCS containing 250 μg/ml tetrazolium bromide, M2128-Sigma) was added to each well. Cells were further incubated for 90–120 min at 37 °C. Medium was aspirated, and tetrazolium crystals were dissolved by adding 100 μL isopropanol to each well. Absorbance at 490 nm was determined using an ELISA reader (BioTek). To determine the CC_50_, MTT values were calculated in percentage with the respective DMSO control set as 100 %. CC_50_ values were computed by non-linear regression analysis using GraphPad Prism 9.0 (GraphPad Software). WST-1 assays (water-soluble tetrazolium assay) were performed to determine the toxicity of PatA and DMDAPatA in HepG2 cells. The CC_50_ values of PatA and DMDAPatA were determined by incubating 2 × 10^4^ HepG2 cells per well with serial dilutions (0 nM, 0.1 nM, 0.5 nM, 1.0 nM, 10 nM, 50 nM and 100 nM) of the corresponding compound in a 96-well plate (Cellstar® 96-well Microplate, flat bottom clear polystyrene wells, Greiner Bio-One). After 48 h of incubation, WST-1 reagent was diluted 1:11 in PBS, and 110 μL was added to each well. After 2 h of incubation at 37 °C and 5 % CO_2_, cytotoxicity was measured in a Tecan microplate reader (Tecan Infinite M Plex).

### 2.5 Antiviral activity

To determine the antiviral activity of pateamines, MRC-5 cells were infected with HCoV-229E or MERS-CoV at an MOI of 0.1 for 1 h [12]. After incubation, the inoculum was removed and cells were incubated for 24 h with fresh medium containing PatA or DMDAPatA at different concentrations (100 nM, 50 nM, 10 nM, 5 nM, 1 nM, 0.5 nM, 0.1 nM). After incubation, supernatant was collected and plaque assay was performed to analyze virus titer. Briefly, cells were seeded in 24-well plates and inoculated for 1 h with 10-fold serial virus dilutions in DMEM with 10 % FCS. Virus inoculum was removed and Avicel-containing medium (1x MEM (Gibco), 1.25 % Avicel (FMC Biopolymer)) was added. At 24 h p.i., the plates were washed with PBS, fixed with 3.7 % PFA in PBS and the cell layer was stained with 0.15 % crystal violet. To calculate EC_50_ values, the virus titer determined for virus-infected untreated cells was set to 100 % and titers obtained for PatA- or DMDAPatA-treated cells were calculated in relation to untreated control. The EC_50_ values were calculated by non-linear regression analysis using GraphPad Prism 9.0 (GraphPad Software).

### 2.6 Software

For sequence analysis and primer design, SnapGene® software (from Dotmatics; available at snapgene.com) was used. GraphPad Prism version 9 for Windows, (GraphPad Software, Boston, Massachusetts USA) was used for data and statistical analysis as well as to design the graph reported in the present work. For protein purification, UNICORN™ (Version 6.0) was used on an ÄKTA pure system (GE Healthcare). Protein and ligand preparation for docking analysis, as well as energy minimization, were performed using the Molecular Operating Environment (MOE) (2022.02 Chemical Computing Group ULC, 910–1010 Sherbrooke St. W., Montreal, QC H3A 2R7, Canada, 2023). Docking analysis was performed using the Genetic Optimization for Ligand Docking (GOLD) [25]. The PyMOL Molecular Graphics System (Version 2.0, Schrödinger, LLC) was used to both, analyze the docking poses and for picture generation.

## 3 Results

### 3.1 Mode of action of pateamines

In previous studies, we and others have investigated the antiviral activity and mode of action of rocaglates *in vitro* and *in vivo* [11,12,15]. Apart from rocaglates, pateamines are also known to be efficient, but less characterized eIF4A inhibitors. Therefore, we analyzed (i) the relevance of different amino acids for RNA clamping by pateamines, (ii) the dependency for a polypurine tract in the RNA substrate to allow efficient RNA clamping, and (iii) the antiviral activity of pateamines against coronaviruses.

**Fig. 1:**
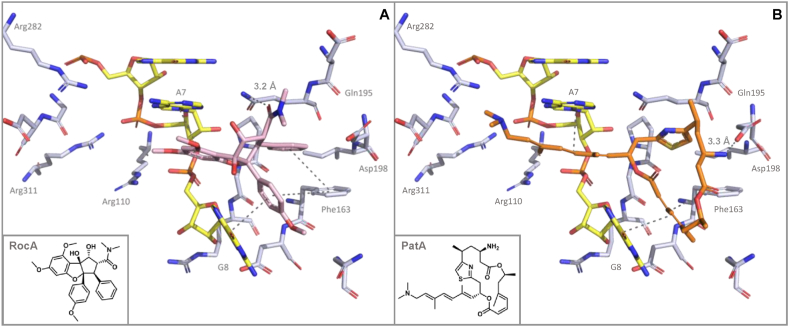
Comparison between the binding mode of **A)** the rocaglate Rocaglamide A (RocA, PDB: 5ZC9) and **B)** the docking pose of the pateamine Pateamine A (PatA) in complex with the polypurine RNA (AG)_5_ (yellow sticks with phosphorus atoms colored orange) in the RNA binding pocket of eIF4A (grey stick - nitrogen atoms in blue. oxygen atoms in red). RocA is depicted as a pink and PatA as an orange stick model. Main important interactions are highlighted as grey dashes. The same color codes, sticks, dashes and surface representations are also applied in Suppl. Fig. S1.

In a first attempt, we compared the binding mode of the rocaglate RocA with that of pateamines by docking PatA into the published eIF4A-(AG)_5_-RocA crystal structure (Fig. 1A, [22]). As shown in Fig. 1B, PatA cannot form a stable π-π stacking interaction with phenylalanine F163 (position based on human eIF4A sequence) as it was described for RocA. We have recently shown that F163 is a crucial amino acid for rocaglate binding to eIF4A-RNA complexes and that amino acid variation at this position determines rocaglate-sensitivity or -resistance in eukaryotes [19]. Interestingly and in contrast to RocA, the primary amino group of PatA may form a hydrogen bond (3.3 Å) with the carboxylic group of aspartate D198, an amino acid that is highly conserved in eIF4A. Docking of DMDAPatA revealed that this compound cannot form an H-bond with D198 due to the missing amino group (Suppl. Fig. S1). The interaction of pateamines with the purines A7 and G8 in the bound (AG)_5_ RNA substrate seems to be similar to that observed for RocA.

**Fig. 2:**
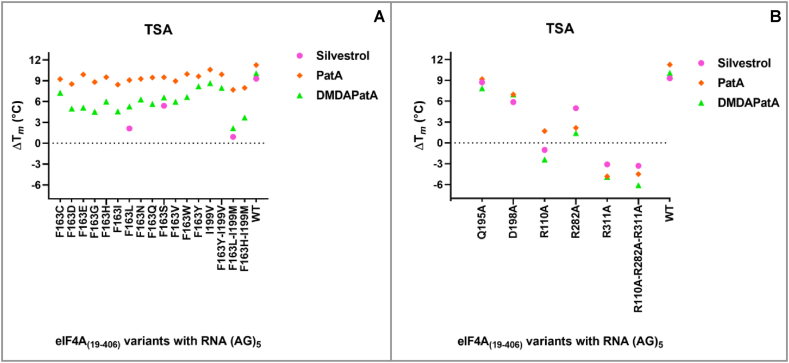
**A-B)** Thermal Shift Assay (TSA) plot of different eIF4A_(19-406)_ variants with the polypurine RNA (AG)_5_ and the non-hydrolyzable ATP analogue adenylyl imidodiphosphate (AMP-PNP) in complex with the PatA (orange), the synthetic desmethyl-desamino-PatA (DMDAPatA in green) and silvestrol (magenta) for comparison. **A)** ΔT_*m*_ of 13 different mutants at position F163, as well as I199V and three double mutants compared to the WT. As previously described [19], rocaglates like silvestrol need phenylalanine, tyrosine, histidine or valine at amino acid position 163 (human eIF4A) to bind and clamp the eIF4A-(AG)_5_ complex. For better clarity, ΔT_*m*_ of silvestrol is only shown for the eIF4A variants found in *Aglaia* (F163I, F163L-I199 M)*, Leishmania* (F163S) and for the human eIF4A sequence (WT). Contrary, PatA binding is independent from amino acid variations at position 163 since ΔT_*m*_ does not change among the different mutants. For DMDAPatA a substantial drop in ΔT_*m*_ (up to 5 °C for single mutants and up to 8 °C for double mutants) can be observed. **B)** ΔT_*m*_ of relevant amino acid variants within the RNA binding pocket as well as the arginine pocket [19]. Q195A seems not to play an important role in the pateamine binding while D198A showed a reduction of about 5 °C compared to the WT. ΔT_*m*_ were compared to the DMSO control for n ≥ 3. For ΔT_*m*_ and SEM values see Supplementary Table 1 (SI).

Based on this *in silico* comparison, we started to systematically analyze the binding of pateamines to a total of 23 different eIF4A variants (19 with single mutations, three with double mutations and one with a triple mutation) using TSA to gain information on eIF4A-RNA complex stability in the presence of eIF4A inhibitors by analyzing ΔT_*m*_ values. All eIF4A variants were overexpressed and purified as functional enzymes with RNA helicase activity (Suppl. Fig. S2. [19]). As an RNA substrate, we used the (AG)_5_ polypurine sequence and as a control to evaluate TSA outcome, we included the rocaglate silvestrol. For silvestrol, melting temperatures were strongly reduced as exemplarily shown for the F163L mutant (∼9.5 °C) and for the double mutant F163L-I199 M (nearly 9 °C) responsible for rocaglate-resistance in *Aglaia* (Fig. 2A, pink dots; see also [19]). The ΔT_*m*_ during PatA treatment were rather slight (∼1–2.5 °C) still leading to ΔT_*m*_ levels comparable to the ΔT_*m*_ observed for silvestrol treatment in wildtype eIF4A. Even in the double mutant F163L-I199 M the increase in T_*m*_ is still 7.7 °C, thus PatA binding, and RNA clamping seems to be independent of F163. To prove this assumption, we tested the effect of PatA treatment against *Leishmania major* since eIF4A from this pathogen contains a serine at position F163 which makes *Leishmania* rocaglate-resistant [19]. As shown in Fig. 2A and Table 1, silvestrol can bind to the F163S variant, however, the ΔT_*m*_ is only 5.4 °C. This might indicate that stable RNA clamping requires ΔT_*m*_ levels >5.4 °C. Importantly, in the presence of PatA thermal stability of the eIF4A-RNA complex increases by about 9.5 °C. Indeed, in contrast to rocaglates, treatment of *Leishmania major* promastigotes with PatA leads to antipathogenic activity with an EC_50_ of ∼550 nM (Suppl. Fig. S3).

For DMDAPatA the situation is different. Here, the drop in ΔT_*m*_ is more pronounced (up to 5.5 °C for single mutants and about 8 °C for F163L-I199 M), thus DMDAPatA binding and RNA clamping relies on F163. Apart from F163, we also mutated glutamine Q195 and D198 to alanine (A) since they might be relevant for rocaglate or pateamine binding via hydrogen bonds. For Q195A the effects were again rather mild (∼2 °C drop in T_*m*_ for pateamines, ∼1 °C drop in T_*m*_ for silvestrol) whereas for D198A the ΔT_*m*_ were more pronounced (∼3–4 °C) demonstrating that D198 has some relevance for proper rocaglate and pateamine binding. Since this amino acid is highly conserved, it might have a general influence on the surface structure of eIF4A. However, a hydrogen bond with D198 can only be formed by PatA. This might explain the 1–1.5 °C higher ΔT_*m*_ value during PatA treatment compared to DMDAPatA and silvestrol. Next, we mutated three arginines (R110, R282 and R311) to alanines since they form an arginine pocket that is important for proper RNA binding [19]. For all arginine mutants, melting temperatures were strongly affected (R311A > R110A > R282A) in a similar manner for all tested compounds, thus showing that correct binding of the RNA substrate is a prerequisite for efficient RNA clamping.

### 3.2 Pateamines do not require a polypurine stretch for RNA clamping

**Fig. 3:**
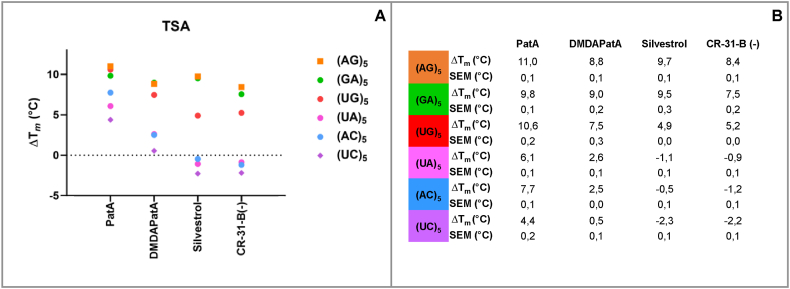
**A)** Effect of different RNA oligos (10mers) on inhibitors binding to eIF4A WT. A polypurine stretch promotes the inhibitor's binding while a fully polypyrimidine stretch discourages the binding. ΔT_*m*_ are compared to the DMSO control for n ≥ 3. **B)** ΔT_*m*_ and SEM values for the different eIF4A-RNA inhibitors.

Based on the crystal structure of the eIF4A-(AG)_5_-RocA complex [22] and reporter gene assays [12], it was proposed that RNA clamping by rocaglates depends on π-π stacking interactions between a rocaglate and two consecutive purines embedded in a polypurine tract. From our docking pose (see Fig. 1B), we claimed a similar mechanism for RNA clamping by pateamines. To test this, we analyzed six different RNA 10mer oligonucleotides consisting of purines ((AG)_5_, (GA)_5_), mixed sequences of purines and pyrimidines ((UG)_5_, (UA)_5_, (AC)_5_) and only pyrimidines ((UC)_5_) as substrates in TSA. We determined the ΔT_*m*_ values for treatment with PatA, DMDAPatA, silvestrol and the synthetic rocaglate CR-31-B (−) [12]. As expected, both rocaglates show strong binding in the presence of polypurines. However, in the presence of the mixed sequence (UG)_5_, rocaglates still show some binding by increasing the T_*m*_ to about 5 °C. Interestingly, the mixed (UA)_5_ and (AC)_5_ oligos as well as the pyrimidine oligo (UC)_5_ cannot promote any binding of rocaglates to eIF4A (Fig. 3A). Regarding PatA, we observed ΔT_*m*_ values of 10–11 °C for the purine oligos and, surprisingly, also for the mixed (UG)_5_ sequence. This implies that guanine is required to mediate efficient binding of PatA that can be surrounded by pyrimidines (Fig. 3A). Moreover, the mixed sequence (AC)_5_ still facilitates an increase in T_*m*_ of 7.7 °C. Only the pyrimidine oligo mediates an increase in T_*m*_ of less than 5 °C (see ΔT_*m*_ values Fig. 3B). With DMDAPatA we found similar effects in the presence of purine oligos or (UG)_5_. Interestingly, guanines seem to be crucial, since no binding of DMDAPatA could be observed in the presence of (UA)_5_, (AC)_5_ or (UC)_5_ oligonucleotides.

### 3.3 Effects of pateamines on translation initiation

**Fig. 4:**
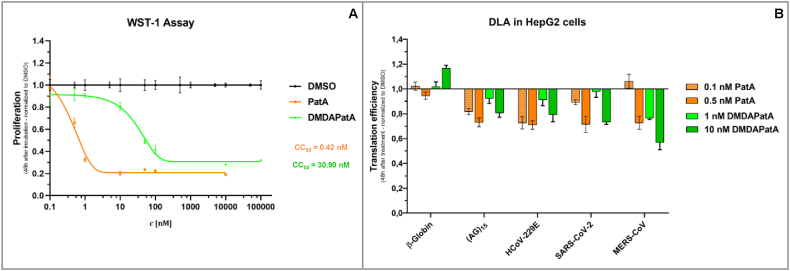
**A)** Cytotoxic Concentration _50_ (CC_50_) calculation of PatA and DMDAPatA in HepG2 cells. **B)** Dual-Luc-Assay (DLA). Effects of PatA and DMDAPatA on translation in the presence of 5′-UTRs originating from coronaviruses HCoV-229E, SARS-CoV-2 and MERS-CoV.

Next, we investigated the effects of pateamines on translation initiation in the cancer cell line HepG2 by using a dual luciferase reporter assay (DLA). For this, we first determined the cytotoxicity of PatA and DMDAPatA by measuring their effects on cell proliferation in a WST-1 assay. Cytotoxicity in cancer cell lines for the inhibition of eIF4A is generally expected since rocaglates and pateamines are originally described as compounds with potent anticancer activity by blocking cell proliferation at low nanomolar concentrations [1,26,27]. Therefore, we also expected potent effects of pateamines on cell viability in HepG2 cells. As shown in Fig. 4A, the CC_50_ value for PatA was 0.42 nM and for DMDAPatA 30.90 nM. Based on this, we performed the DLA at 0.1 or 0.5 nM concentrations for PatA as well as 1 and 10 nM concentrations for DMDAPatA. As a negative control, we used the 5′-UTR of the β-globin mRNA because it is rather unstructured, and translation is eIF4A independent [28]. Both pateamines did not reduce translation efficiency in the presence of the β-globin 5′-UTR, which is in line with the effects observed for rocaglates [28]. As expected, the (AG)_5_ polypurine sequence promotes RNA clamping and therefore a reduction in translation efficiency (Fig. 4B). Next, we tested the effects of both pateamines on 5′-UTRs from HCoV-229E, SARS-CoV-2 and MERS-CoV in the DLA. In all cases, we measured a reduction of translation efficiency in the presence of pateamines in a dose-dependent manner. However, the reduction of translation efficiency by PatA mediated via the 5′-UTR of HCoV-229E could not be increased at the higher PatA concentration (Fig. 4B). In summary, the DLA points to the possibility that pateamines might also be active against different coronaviruses in infected cells.

### 3.4 Potent antiviral activity of pateamines in coronavirus-infected cells

**Fig. 5:**
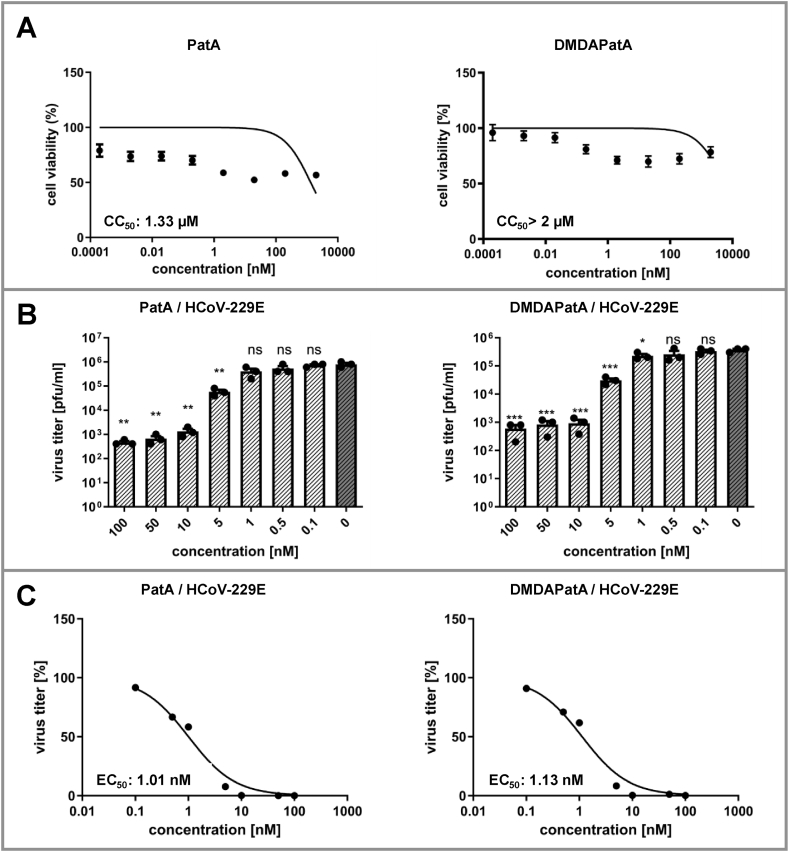
Determination of cytotoxicity and antiviral activity of PatA and DMDAPatA in MRC-5 cells. **A)** Analysis of cytotoxicity of PatA and DMDAPatA. Effects on cell proliferation were measured by an MTT assay. MRC-5 cells were incubated with the indicated concentrations of the compounds and MTT assays were performed after 24 h. Results were based on three independent experiments (n = 3). **B)** and **C)** Determination of virus titers after treatment with PatA or DMDAPatA. MRC-5 cells were infected at an MOI of 0.1 with HCoV-229E for 24 h in the presence of indicated concentrations of PatA or DMDAPatA. Virus titers were determined using plaque assays. Significance levels compared to the results for untreated cells are indicated as follows: *. p < 0.05; **. p < 0.005; ***. p < 0.0005. Error bars show SD. **C)** Virus titer reduction (in percentage) was calculated in relation to infected controls without compound treatment (mock), and EC_50_ values were calculated using non-linear regression analysis. Results were based on three independent experiments (n = 3).

In the last set of experiments, we investigated the antiviral effects of pateamines against HCoV-229E and MERS-CoV in primary human fetal lung fibroblasts (MRC-5 cells) as a well-established cell system for infection with these two coronaviruses [29]. First, we tested cytotoxicity of pateamines in MRC-5 cells 24 h post-treatment using MTT assay. At low nanomolar concentrations, cell viability was slightly affected, however, the calculated CC_50_ values for both compounds were >1 μM (Fig. 5A), demonstrating no major cytotoxic effects of pateamines in MRC-5 cells. Importantly, at a concentration of 10 nM HCoV-229E virus titers were reduced by about three log phases (Fig. 5B) and corresponding EC_50_ values of about 1 nM could be determined for PatA and DMDAPatA (Fig. 5C). For MERS-CoV virus titers were reduced about 3.5 log phases with PatA at a concentration of 50 nM and the corresponding EC_50_ value was determined at 7.62 nM (Suppl. Fig. S4). For DMDAPatA MERS-CoV titers were reduced about 2.5 log phases at 100 nM (Suppl. Fig. S4, panel A) and the EC_50_ value was determined at a concentration of 39.48 nM (Suppl. Fig. S4, panel B). From our data, we concluded that PatA and DMDAPatA have potent anti-coronaviral activity at low nanomolar concentrations, although PatA seems to be somewhat more active in MERS-CoV-infected cells.

**Fig. 6:**
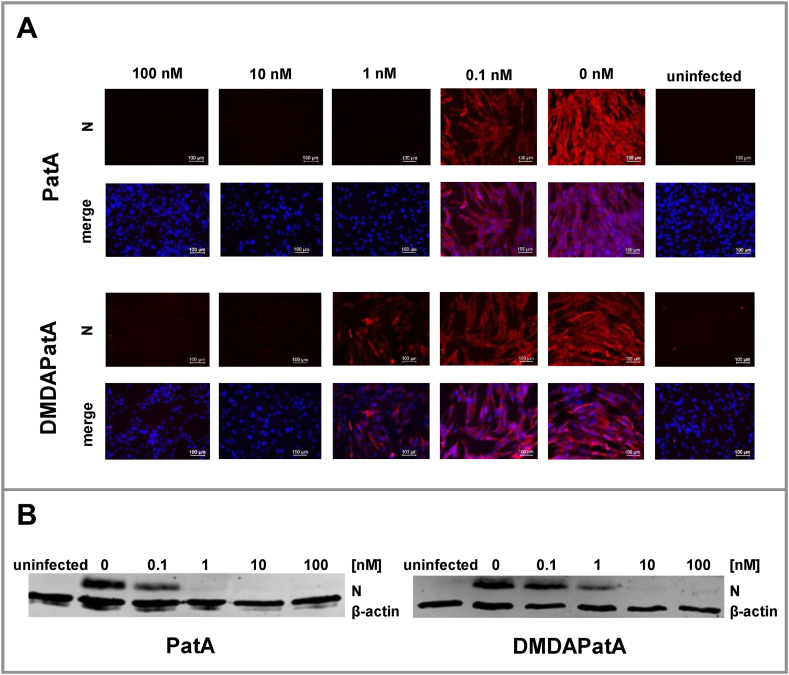
Analysis of viral nucleoprotein (N) expression after treatment with PatA or DMDAPatA in MRC-5 cells. **A)** Immunofluorescence analysis or **B)** Western blot analysis to visualize the effects of pateamines on viral N protein accumulation in HCoV-229E-infected MRC-5 cells. For presentation of full, non-adjusted images of the shown western blots see Suppl. Fig. S5. Cells were infected at an MOI of 1, incubated with the indicated compound concentrations for 24 h p.i. and analyzed **A)** by microscopy or **B)** western blotting using specific antibodies for viral N protein (n = 3). Blue: DAPI-stained cell nuclei. Red: N protein. Shown are representative data from at least three independent experiments.

Finally, we investigated the effects of pateamines on viral protein accumulation in infected MRC-5 cells by analyzing the nucleoprotein (N protein) levels using immunofluorescence or western blotting. Reduced N protein levels could be observed at 0.1 nM for PatA treatment. At a concentration of 1 nM no N protein could be detected (Fig. 6 A, B). In comparison, treatment of MRC-5 cells with DMDAPatA at a concentration of 1 nM reduced N protein levels and at a concentration of 10 nM DMDAPatA the N protein levels were reduced to undetectable amounts.

Taken together, the RNA clamping by PatA is independent of π-π stacking interactions with an amino acid at position 163 and does not require a polypurine tract. Nevertheless, guanine in the RNA substrate strongly promotes RNA clamping of pateamines. In addition, both pateamines show potent anti-coronaviral activity whereby PatA seems to be more toxic.

## 4 Discussion

In this study, we discovered that pateamines are potent anti-coronaviral compounds, significantly reducing the titers of HCoV-229E and MERS-CoV by around three log phases at low nanomolar concentrations in MRC-5 cells. As previously known, pateamines exhibit high toxicity in cancer cell lines, while their cytotoxicity in human fetal lung fibroblasts (MRC-5), as a model cell system for coronaviral infection, is rather low. Similar to rocaglates, pateamines target the RNA helicase eIF4A through a mechanism known as RNA clamping, thereby interfering with the translation initiation of specific cellular and viral mRNAs. This dependency on eIF4A necessitates stable RNA structures in the 5′-UTRs of mRNAs that need to be unwound during translation initiation. Additionally, RNA clamping requires stacking interactions with purines. These stable RNA structures, combined with polypurine tracts, are frequently found in mRNAs from proto-oncogenes and viral mRNAs. Moreover, ribosome profiling experiments have revealed that only approximately 300 mRNAs are eIF4A-dependent [30] (own unpublished data). This may explain why pateamines and rocaglates exhibit potent antiviral and anticancer activities without causing significant toxicity in a broad range of normal healthy cells. In line with this, rocaglates have already been characterized as compounds with a promising safety profile in preclinical studies [31,32].

Importantly, crystal structures for RocA and desmethyl-PatA binding to an eIF4A-(AG)_5_ complex are available in the Protein Data Bank (PDB ID: 5ZC9, 6XKI), revealing binding of both to the same pocket in a comparable manner. From these structural data and from biochemical analysis [23] it has been proposed that at least three molecular interfaces are relevant for RNA clamping by rocaglates and pateamines, namely π-π stacking interactions of the compounds with F163 and additional two π-π stacking interactions with purines in the bound RNA substrate. Interestingly, both compounds are natural molecules originating from plants or a marine sponge, respectively, thus two structurally different molecular scaffolds (a cyclopenta[*b*]benzofurane for rocaglates and the thiazole-containing macrolide PatA) have emerged in two completely different organisms to inhibit eIF4A, which is a remarkable example of molecular mimicry during evolution. It has been speculated that rocaglates protect *Aglaia* plants from insects or other pathogens. This hypothesis was supported by the fact that *Aglaia* sp. have an eIF4A variant with the mutation F163L, preventing RNA clamping by rocaglates and leading to rocaglate-resistance. Importantly, the fungus *Ophiocordyceps* sp., which can be found on *Aglaia* plants, has also the eIF4A variant F163L [33]. Unfortunately, no sequence information regarding eIF4A from *Mycale hentscheli* is currently available.

In our study, we discovered that RNA clamping by PatA is independent of F163, Q195, and D198, suggesting that the macrocycle of the macrolide can occupy the binding pocket on eIF4A without the need to interact with specific amino acids. However, DMDAPatA, which cannot form a hydrogen bond with the carboxylic group of D198, exhibits a modest reduction in thermal shift of 1.2 °C compared to PatA. This reduction indicates some stabilizing effect of D198, although it is not a prerequisite for RNA clamping.

Nonetheless, our findings do not match with those of Naineni and colleagues, who observed a dependency of RNA clamping by desmethyl-PatA (DMPatA) on F163, Q195, and D198, using a fluorescence polarization assay [23]. The variance, particularly for D198, may be attributed to the different eIF4A variants used. In our study, we introduced a neutral mutation to alanine (D198A), whereas in the study by Naineni and colleagues, a positively charged lysine was introduced (D198K). Regarding the eIF4A variants F163L and F163S, our data with DMDAPatA show similar results to those observed with DMPatA. To further elucidate the significance of F163 for RNA clamping with PatA, we conducted an experiment using *Leishmania major* promastigotes that harbor a serine instead of a phenylalanine at the relevant eIF4A position. Indeed, promastigotes displayed susceptibility to PatA treatment (refer to Fig. S3), supporting our hypothesis that PatA can engage in RNA clamping independently of the chemical properties of the amino acid located at position 163 by filling the binding pocket space via its macrocycle.

Upon analyzing RNA substrates, we observed that PatA can, in principle, clamp pyrimidine-containing substrates maybe by forming a linear π-conjugated system that interacts with all four bases. However, the presence of the purine base guanine strongly enhances the efficiency of RNA clamping. In the case of DMDAPatA and rocaglates, it appears that RNA clamping with pyrimidine tracts or mixed purine/pyrimidine sequences lacking guanine is not feasible. Once again, the inclusion of guanines raises the melting temperature by more than 5 °C, underscoring the essential role of guanines in the RNA substrate for effective RNA clamping.

Targeting a host factor like eIF4A offers several advantages, including broad antiviral activities [29] and immunomodulatory effects [34,35]. It is rather unlikely that viruses can develop strategies to escape host targeting since they can only mutate their own genome. In line with this, we have already demonstrated the unlikelihood of viral escape mutants emerging during rocaglate treatment [36]. Pateamines have also exhibited broad antiviral activities against Sindbis-, Zika-, and Influenza A viruses [2,3,16], in addition to coronaviruses as shown in this study. These findings underscore eIF4A as a promising target for developing pan-antivirals urgently needed during outbreak situations involving newly emerging viruses.

Taken together, our results show that pateamines broaden our toolbox for combating viruses that rely on the host enzyme eIF4A for conducting their viral protein synthesis. In addition, we demonstrated that pateamines can serve as effective eIF4A inhibitors in rocaglate-resistant pathogens. Our results indicate a possible treatment approach of pateamines against various current and emerging coronaviruses or other viruses with eIF4A-dependent translation. Since pateamines are host-directed antivirals, cytotoxic effects on the organism must be expected when applied systemically. Therefore, topic application via aerosol (instead of a systemic application) can reduce the risk of potential toxic side effects and eliminate the first-pass effect of the liver. Thus, a local application of pateamines for future *in vitro* and *in vivo* studies should be considered.

## Financial support

This research was funded by the LOEWE Center DRUID (projects A2, B2 and D3, to A.G., J.Z. and G.v.Z.), the 10.13039/100009139German Center for Infection Research (10.13039/100009139DZIF), the partner site, Giessen-Marburg-Langen (TTU Emerging Infections, to J.Z.), the 10.13039/501100002347BMBF project HELIATAR (A.G., A.H. and J.Z) and the GRK2581 (project 10, to J.Z.). The work was also supported by a Research Grant of the University Medical Center Giessen and Marburg (10.13039/501100009560UKGM, to C.M.-R.), the von Behring-Röntgen Stiftung (project 71_0016, to C.M.-R.) as well as the Deutsche Forschungsgemeinschaſt (10.13039/501100001659DFG, project 530813989, to C.M.-R.).

## Data availability

Data associated with this study has not been deposited into a publicly available repository. All relevant data are included in the article or in the supp. material and are referenced in the article. Additional data can be provided upon request.

## CRediT authorship contribution statement

**Francesca Magari:** Writing – review & editing, Writing – original draft, Visualization, Validation, Software, Methodology, Investigation, Formal analysis, Data curation, Conceptualization. **Henri Messner:** Validation, Methodology, Investigation, Formal analysis, Data curation. **Florian Salisch:** Validation, Methodology, Investigation, Formal analysis, Data curation. **Stella M. Schmelzle:** Writing – original draft, Methodology, Investigation, Formal analysis, Data curation. **Ger van Zandbergen:** Supervision, Funding acquisition, Data curation, Conceptualization. **Alois Fürstner:** Resources, Formal analysis, Conceptualization. **John Ziebuhr:** Supervision, Funding acquisition, Conceptualization. **Andreas Heine:** Supervision, Funding acquisition. **Christin Müller-Ruttloff:** Writing – review & editing, Writing – original draft, Visualization, Validation, Supervision, Resources, Project administration, Methodology, Investigation, Funding acquisition, Formal analysis, Data curation, Conceptualization. **Arnold Grünweller:** Writing – review & editing, Writing – original draft, Validation, Supervision, Project administration, Methodology, Investigation, Funding acquisition, Formal analysis, Data curation, Conceptualization.

## Declaration of competing interest

The authors declare that they have no known competing financial interests or personal relationships that could have appeared to influence the work reported in this paper.
