# Supplementary material for: Potent anti-coronaviral activity of pateamines and new insights into their mode of action
Source: Heliyon. 2024 Jun 22;10(13):e33409. doi: 10.1016/j.heliyon.2024.e33409 (PMC11259845; doi:10.1016/j.heliyon.2024.e33409)
Supplement: Multimedia component 1 [file mmc1.docx]

**Supporting Information (SI):**

**Supplementary materials and methods**

*1.1 Site-directed mutagenesis, protein expression and purification*

eIF4A variants were produced using PCR-based site-directed mutagenesis. As template the plasmid encoding for the *human* eIF4A1 pET-28a(+)_eIF4A1_(19-406)_ was used. Single-point mutations were introduced by designing respective forward and reverse primer carrying the desired mutation. The plasmids consisted of a T7 promoter, an N-terminal 6xHis-Tag, a thrombin cleavage site and a resistance gene against Kanamycin. After DpnI digestion, the product was transformed into *E. coli* TOP10 competent cells and the purified plasmids were sequenced to confirm the corresponding mutations.

For protein expression, competent *E. coli* cells (strain BL21(DE3)) and the corresponding plasmid were carefully mixed. After 30 min of incubation on ice, cells were treated by a heat-shock for 30 sec at 42 °C. The transformation reaction was grown in LB media at 37 °C for one hour and subsequently plated on agar plate containing the antibiotic Kanamycin at a final concentration of 50 µg/mL. The agar plate was incubated at 37 °C overnight. The next day, a single colony was picked from the agar plate and transferred into 100 mL of LB medium and 100 μL of Kanamycin. This pre-culture was incubated at 37 °C in a shaking incubator (MINITRON. INFORS HT) at 180 rpm overnight. Afterwards, 10 mL of the pre-culture was transferred into 500 mL of LB media containing 500 µL Kanamycin (50 µg/mL). Protein expression was induced at OD_600_ value of 0.5-0.6. Therefore, the temperature was reduced to 16 °C and IPTG was added to a final concentration of 0.5 mM. The cell culture was incubated overnight at 16 °C and 180 rpm in a shaking incubator (MINITRON. INFORS HT). The cell culture was then centrifuged for 45 min at 10000 rpm at 4 °C (Centrifuge 5810R, Eppendorf). After removal of the supernatant, the cell pellet was collected and stored at -80 °C.

The pellet was resuspended in lysis buffer consisting of 20 mM HEPES–KOH buffer pH 7.5, 300 mM KCl, 20 mM imidazole, 5 mM β-mercaptoethanol, 10% (v/v) glycerol, Lysozyme (50µg/mL) and Benzonase (Benzonase® Nuclease, 5KU, Sigma-Aldrich/Merck). One cOmplete™ Mini Protease Inhibitor Cocktail, EDTA-free Tablet (Roche. Germany) was added to the solution. The cells were lysed using a sonicator (SONIFIER 250, BRANSON) and then centrifugated at 10000 rpm at 4 °C for 60 min (Avanti™ J25. BECKMAN COULTER™). The supernatant was then collected and filtrated with a 0.45 μm filter (Ultrafree®-MC filters, pore size 0.45 μm, Merck), loaded onto a 5 mL Ni-NTA column (HisTrap HP, GE Healthcare Life Science. Freiburg, Germany) and eluted (2 mL/min, 0.5 mPa) with a buffer consisting of 20 mM HEPES–KOH buffer pH 7.5, 300 mM KCl, 250 mM imidazole, 5 mM β-mercaptoethanol, 10% (v/v) glycerol. The protein elution was performed by applying a liner gradient of imidazole. The eluted protein solution was mixed with thrombin protease (10 µg of thrombin per mg of protein) to cleave the 6xHis-Tag, transferred in a dialysis membrane (Regenerated Cellulose Tubular Membrane MWCO: 12000-14000. Roth™) and dialyzed overnight at 4 °C against 4 L of buffer (20 mM HEPES–KOH buffer pH 7.5, 300 mM KCl, 5 mM MgCl_2_, 1 mM DTT and 10% (v/v) glycerol). To remove the protease as well as putative unspecific proteins, the sample was loaded on another 5 mL Ni-NTA column and the flow through containing the targeted protein was collected and concentrated by centrifugation (Heraeus® Multifuge® 3SR, Thermo Scientific) to a volume of 10 mL using an Amicon concentrator with 10 kDa cut-off (Amicon®, 20 mL, MWCO 10000, Sartorius Stedim Biotech GmbH). The thrombin protease was removed via a pAmino-Benzamidine column by applying an increasing concentration of ionic strength (20 mM HEPES–KOH buffer pH 7.5, 1 M KCl, 1 mM DTT and 10% (v/v) glycerol). As an additional purification step, a size exclusion chromatography was performed. The protein was loaded onto a SEC column (Superdex 200, GE Healthcare Life Science, Freiburg, Germany) and eluted with a running buffer consisting of 20 mM HEPES–KOH buffer pH 7.5, 300 mM KCl, 5 mM MgCl_2_, 1 mM DTT and 10% (v/v) glycerol. The protein solution was concentrated (Heraeus® Multifuge® 3SR, Thermo Scientific) to 4 mg/mL, flash frozen in liquid nitrogen and stored at -80 °C.

*1.2 RNA oligonucleotides*

RNA oligonucleotides were purchased by Microsynth AG (Balgach, Switzerland) and Biomers.net GmbH (Ulm/Donau, Germany).

| **RNA oligo** | **Sequence** | **Purchased** |
| --- | --- | --- |
| 10mer-Cy3 | 5′-[CY3]GCUUUCCGGU-3′ | Microsynth AG |
| 16mer-BHQ2 | 5′-ACUAGCACCGGAAAGC[BHQ2]-3′ | Microsynth AG |
| 10mer-competitor | 5′-GCU UUC CGGU-3′ | Microsynth AG |
| (AG)_5_ | 5′-AGAGAGAGAG-3′ | Biomers |
| (GA)_5_ | 5′-GAGAGAGAGA-3′ | Biomers |
| (UG)_5_ | 5′-UGUGUGUGUG-3′ | Biomers |
| (UA)_5_ | 5′-UAUAUAUAUA-3′ | Biomers |
| (AC)_5_ | 5′-ACACACACAC-3′ | Biomers |
| (UC)_5_ | 5′-UCUCUCUCUC-3′ | Biomers |

*1.3 Helicase Assay*

Helicase activities of the eIF4A variants were determined using two labelled RNA substrates: a 10mer modified with Cyanine 3 (10mer-Cy3; 5′-[CY3] GCU UUC CGGU-3′). and a 16mer modified with Black Hole Quencher2 (16mer-BHQ2; 5′-ACU AGC ACC GGA AAGC[BHQ2]-3′). An unlabeled competitor (10mer-competitor; 5′-GCU UUC CGGU-3′) was used to capture released quencher RNA. A single-stranded Cy3 RNA substrate (ssRNA) was used to determine the maximum fluorescence signal of the reaction. 1µM of 10mer-Cy3 was mixed with 1µM of 16mer-BHQ2 and the reaction mix was annealed at 80 °C for 5 min to generate the dsRNA and incubated at room temperature for 1 h followed by incubation on ice for 10 min in a 25 mM HEPES (pH 7.4 (KOH)). Competitor RNA was added in 1:10 (v/v) excess to the labelled RNA substrates and the reaction was again incubated on ice for 10 min prior to adding it to the helicase reaction mix. This mix consists of 100 nM of ssRNA or dsRNA diluted in a buffer consisting of 150 mM HEPES-KKOH pH 7.4, 15 mM Mg(CH_3_COO)_2_, 10 mM DTT, 500 mM CH_3_COOK and 1mM ATP. As negative control, two reaction mixes with no protein and no ATP were included. eIF4A (12.5 μM final concentration) was added to the reaction and fluorescence was measured using a TECAN microplate reader (Tecan Infinite M Plex).

*1.4 Dual-luciferase reporter assay (DLA)*

The day before transfection, 2 × 10^4^ HepG2 cells per well were seeded in a 96-well plate (Greiner Bio One International GmbH) in 200 μL IMDM (Lonza) supplemented with 10% FCS (Biochrom AG) and 1% Penicillin/Streptomycin, and incubated at 37 °C at 5% CO_2_. The transfection was performed using 100 μL Opti-MEM (Thermo Fisher Scientific). For the transfection of five wells, 0.5 μg of the reporter construct DNA was diluted in 25 μL of Opti-MEM. In parallel, 0.5 μL of Lipofectamine^®^ 3000 Transfection Reagent (Thermo Fisher Scientific) were mixed with 24.5 μL of Opti-MEM. Both samples were preincubated for 5 min at room temperature, then combined and incubated for another 15 min at room temperature. Then, 10 μL of the solution was added to each well containing the cells in 100 μL Opti-MEM medium. The plates were incubated at 37 °C and 5% CO_2_ and after six h the medium was aspirated and substituted with 200 μL fresh medium (IMDM + 10% FCS + 1% Penicillin/Streptomycin) containing the appropriate PatA (0.1 nM and 0.5 nM) and DMDAPatA (1 nM and 10 nM) concentration. As a control, a solution containing the same DMSO concentration but lacking the corresponding compound was also included. Following incubation of the cells for 48 h at 37 °C at 5% CO_2_, a Dual-Luciferase^®^ Reporter Assay (Promega) was performed according to the manufacturer's instructions. Measurements were done using a Tecan microplate reader (Tecan Infinite M Plex).

*1.5 Western blot analysis*

To analyze viral protein accumulation, MRC-5 cells were infected with HcoV-229E at an MOI of 1. One h p.i., inoculum was removed, PatA or DMDAPatA was added at different concentrations dissolved in DMEM and cells were incubated for 24 h at 33 °C. After incubation, supernatant was discarded and cells were washed with PBS. PBS was replaced with RIPA-Buffer (5M NaCl, 1M Tris-HCL (pH 8.0) 1 mL nonident P-40, 10% sodium deoxycholate, 10% SDS) to collect cells via scraping. After collection, cells were centrifuged and resuspended in RIPA-Buffer containing 1x protease inhibitor for cell lysis. Protein concentration was determined using BCA protein assay kit (Thermo scientific Pierce). Then, Lämmli buffer was added, samples were heated to 95 °C for 5 min for protein denaturation and centrifuged at 13300 rpm for 3 min. Proteins were separated by 10% SDS-PAGE and blotted onto nitrocellulose membrane (Amersham) using transfer buffer (25 mM Tris, 192 mM glycin (pH8.3), 20% methanol (vol/vol).

Membranes were incubated with a Mouse-anti 229E-NP and Rb pAb to detect beta-actin (abcam) respectively, each diluted 1:1000 in PBS containing 3% bovine serum albumin (BSA). Following overnight incubation, the membranes were washed 5x with PBST (1x PBS. Tween-20 (Roth, Germany)) and incubated with appropriate secondary antibodies (IRDye® 680RD Goat anti-Mouse 926-68070 Lot#C80619-05; IRDye® 800CW Goat anti-Rabbit 926-32211 Lot#D20208-05, respectively) diluted 1:15000 in PBS containing 3% BSA. After 2 h, membranes were washed 5x with PBST and analyzed using the LI-COR Odyssey imaging system.

*1.6 Immunofluorescence microscopy*

MRC-5 cells were cultivated on cover slips and infected with HCoV-229E at an MOI of 1 for 1 h. Inoculum was removed and fresh medium with different concentration of either PatA, DMDAPatA or without compound was added. 24 h p.i. cells were fixed with 3.4% paraformaldehyd containing triton X-100 and stained with Mouse-anti 229E-NP antibody. Following overnight staining, cells were washed 3x with PBS and DAPI (1:1000) & Alexa Fluor^TM^ 594 Goat anti-Mouse IgG (1:500) were added for 2 h. Cells were then washed 3x with PBS and images were taken with BZ-X810 (Keyence).

*1.7 Leishmania major*

For virtual screening MHOM/IL/81/FEBNI promastigotes were cultured using NNN blood agar as described (Wenzel et al., 2012). The eIF4A F163S genotype was confirmed by Sanger sequencing (LGC Genomics).

5*10^6^ viable promastigotes from a mid-log growth phase were seeded with increasing concentrations of PatA with 0.5% DMSO (corresponds to DMSO conc. in the 50 µM PatA sample). All conditions were seeded in duplicates.

Parasite viability was assessed using an (3-(4,5-Dimethylthiazol-2-yl)-2,5-diphenyltetrazoliumbromid (MTT; abcam) assay. After 72h of PatA incubation, *L. major* promastigotes were incubated in 1.1mM MTT solution for 4h. Formed formazan crystals were dissolved in DMSO and absorbance was measured at 540nm on a ClarioStar Plus (BMG Labtech).

Absorbance was normalized to the 0.5% DMSO control to yield relative cell viability. IC_50_ determination was conducted via a non-linear regression in GraphPad Prism 9.5.0 (GraphPad Software).

*1.8 Molecular docking*

For virtual screening, the crystal structure of the eIF4A1 in complex with the polypurine RNA (AG)_5_ and RocA (PDB ID: 5ZC9) was obtained from the RCSB Protein Data Bank. Prior docking, the protein structure was prepared in the Molecular Operating Environment (MOE, 2021) by adjusting the charge of protonatable groups and removing water molecules and the ATP analog AMP-PNP. Furthermore, the 3D conformations of the ligands were generated and energy-minimized using MOE. All docking studies were conducted in Genetic Optimization for Ligand Docking (GOLD) (Jones et al. 1997) using the standard setting. The binding pocket was defined as an area of 10 Å far away from the center of the RNA pocket. ASP (Astex Statistical Potential) was used as a scoring function. For each ligand, the 10 docking poses with the highest scoring result were output by the docking program. After post-docking energy minimizations with MOE, the docking poses were visualized and analyzed using PyMOL (Version 2.0. Schrödinger. LLC).

**Figures caption SI**

***S1***: **A)** Superposition of docking pose of PatA (orange stick model) and DMDAPatA (green stick model). D198 is an important residue for PatA binding. In fact, the primary amino group of PatA can form a hydrogen bond (3.3 Å) with the carboxylic group of D198. Interestingly, the D198 changes its orientation to come closer and thus better interact with the amino group of PatA. The same does not happen for the DMDAPatA docked structure, where the macrocycle has a greater flexibility than that of PatA and moves slightly outside the binding pocket. This may be due to both, the lack of H-bond formation (as described above) and the absence of a methyl group, which make the structure of PatA more rigid compared to DMDAPatA, thus increasing the degrees of freedom. **B)** Superposition of the docking pose of PatA (orange stick model) and the crystal structure of RocA (pink stick model. PDB: 5ZC9). The phenyl groups of RocA are involved in perfect π-π stacking interactions, both face-to-face and face-to-edge, with the aromatic ring system of F163. Contrary, only the dienoate and not the thiazole moiety of DMDAPatA is involved in π-π stacking interactions.

***S2***: Helicase assay of the eIF4A mutants Q195A (violet curve) and D198A (pink curve). Both mutants showed a similar helicase activity compared to the WT (green curve). Single-stranded RNA as a positive control (yellow curve) and two negative controls, without ATP (light blue curve) and without protein (red curve), were also measured.

***S3***: Dose-response curve of PatA on *Leishmania major* FEBNI wildtype. PatA was tested for 72h at concentrations of 0nM, 10nM, 100nM, 500nM, 1µM, 5µM, 10µM, 25µM and 50µM with a constant DMSO concentration of 0.5% (corresponds to DMSO conc. in the 50 µM PatA samples). The response for promastigotes in the non-linear fit gives an R^2^ of 0.8999 and an EC_50_ of 0.55 µM. Experiments were done for n=3.

***S4***: **A)** and **B)** Determination of virus titers after treatment with PatA or DMDAPatA. MRC-5 cells were infected at an MOI of 0.1 with MERS-CoV for 24 h in the presence of indicated concentrations of PatA or DMDAPatA. Virus titers were determined using plaque assays. Significance levels compared to the results for untreated cells are indicated as follows: *. p < 0.05; **. p < 0.005; ***. p < 0.0005. Error bars show SD. **B)** Virus titer reduction (in percent) was calculated in relation to infected controls without compound treatment (mock), and EC_50_ values were calculated using non-linear regression analysis. Results were based on three independent experiments (n=3).

***S5***: Presentation of full, non-adjusted western blot images as shown in Fig. 6. Analysis of viral nucleoprotein (N) expression after treatment with PatA (A) or DMDAPatA (B) in HCoV-229E-infected MRC-5 cells. Cells were infected at an MOI of 1, incubated with the indicated compound concentrations for 24 h p.i. and analyzed by western blotting using specific antibodies for viral N protein (n=3).

***Table 1***: Effects of PatA and DMDAPatA addition on the melting temperatures T*_m_* of different eIF4A1_(19-406)_ variants. Tabulation of melting temperatures T*_m_* as well as change in melting temperature ΔT*_m_* compared to the DMSO control with the indication of the standard error of the mean (SEM) for n ≥ 3.

^[1]^: The present values are reported by Obermann et al., 2023.
