# Supplementary material for: Potent anti-coronaviral activity of pateamines and new insights into their mode of action
Source: Heliyon. 2024 Jun 22;10(13):e33409. doi: 10.1016/j.heliyon.2024.e33409 (PMC11259845; doi:10.1016/j.heliyon.2024.e33409)
Supplement: Multimedia component 2 [file mmc2.pdf]

S1

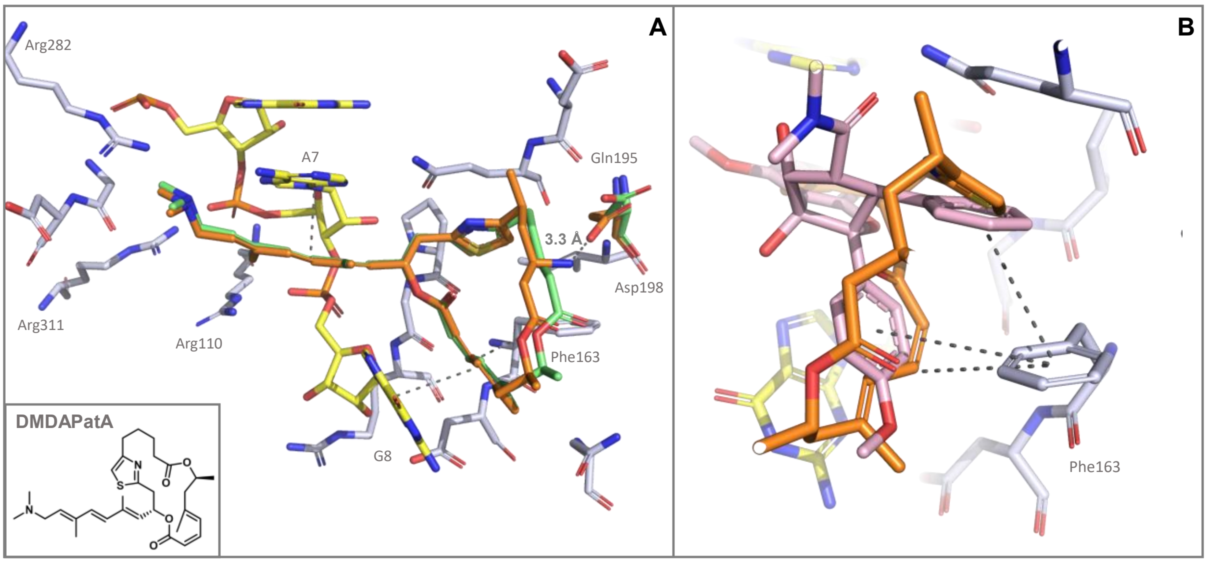
A
B
Arg282
A7
Gln195
3.3 Å
Asp198
Arg311
Arg110
Phe163
G8
DMDAPatA
Phe163

S2

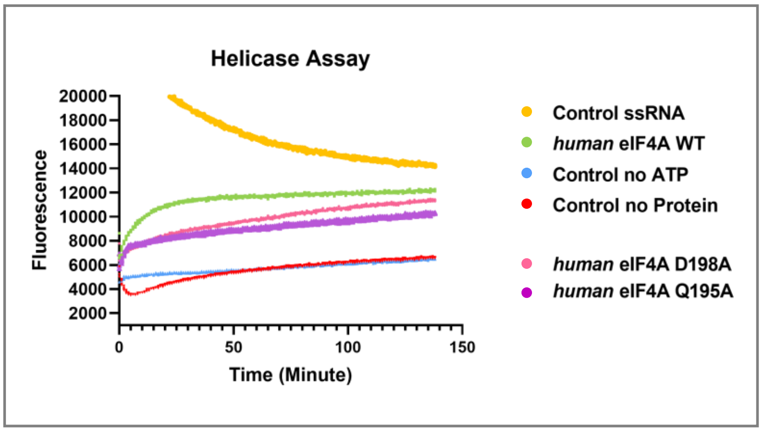
Helicase Assay
Fluorescence
20000
18000
16000
14000
12000
10000
8000
6000
4000
2000
0
50
100
150
Time (Minute)
Control ssRNA
human eIF4A WT
Control no ATP
Control no Protein
human eIF4A D198A
human eIF4A Q195A

S3

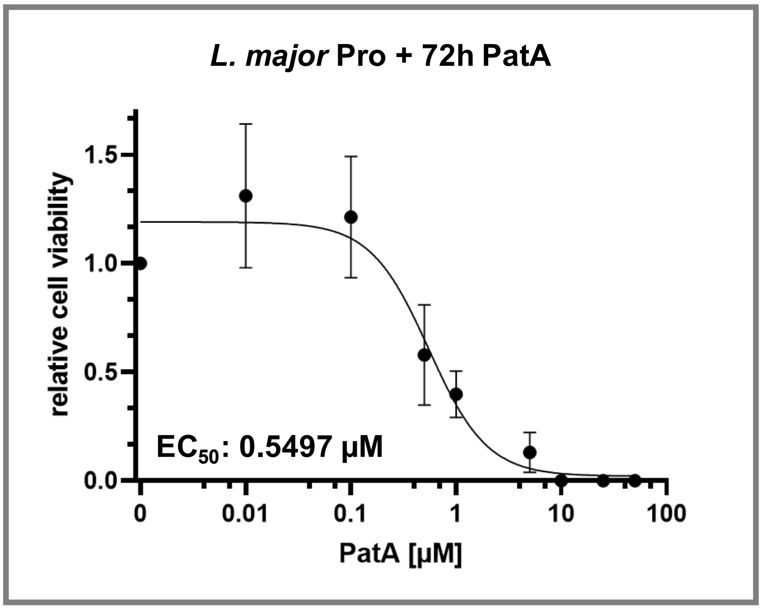

L. major Pro + 72h PatA
relative cell viability
1.5
1.0
0.5
0.0
0
0.01
0.1
1
10
100
PatA [µM]
EC$_{50}$: 0.5497 µM

S4

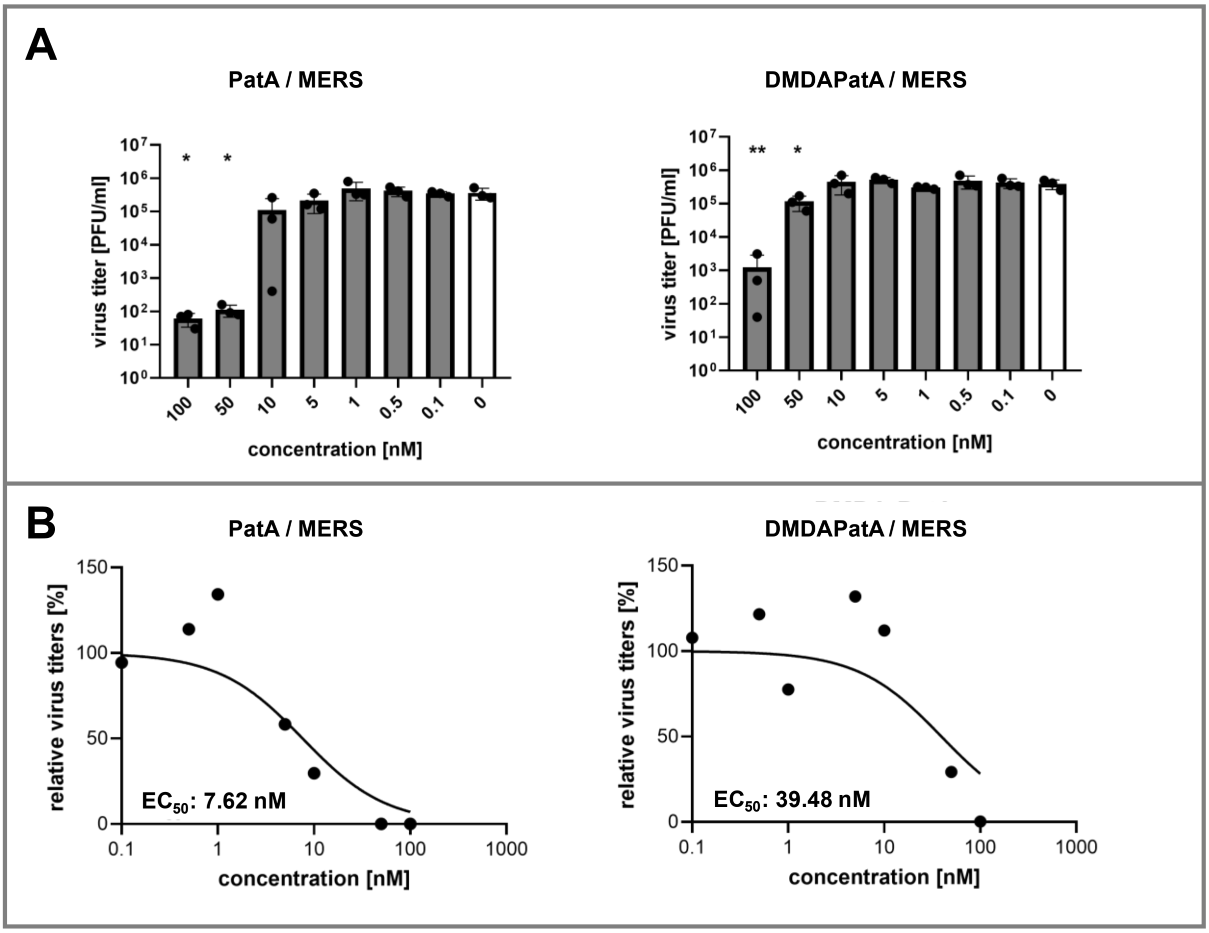

A
PatA / MERS
DMDAPatA / MERS
virus titer [PFU/ml]
10^0 10^1 10^2 10^3 10^4 10^5 10^6 10^7
*
**
100
50
10
5
1
0.5
0.1
0
concentration [nM]
B
PatA / MERS
DMDAPatA / MERS
relative virus titers [%]
150
100
50
0
0.1
1
10
100
1000
concentration [nM]
EC$_{50}$: 7.62 nM
EC$_{50}$: 39.48 nM

S5

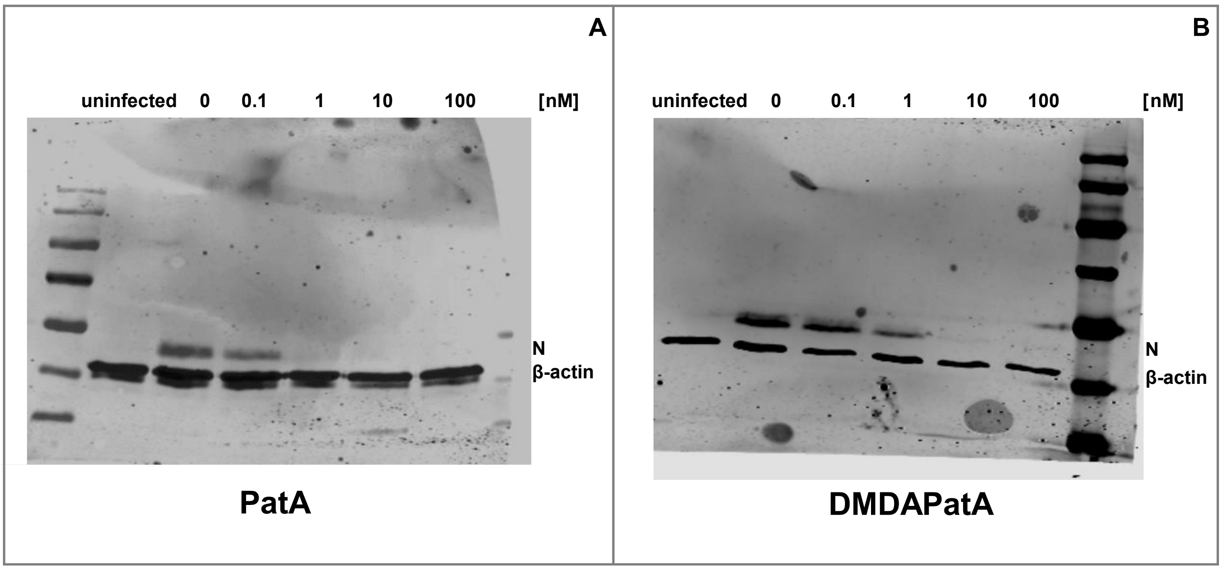
A
uninfected 0 0.1 1 10 100 [nM]
N
β-actin
PatA
B
uninfected 0 0.1 1 10 100 [nM]
N
β-actin
DMDAPatA

Table 1

	PatA		DMDAPatA		Silvestrol[1]	
**eIF4A$_{(19\text{-}406)}$ variant**	**ΔTm [° C]**	**SEM [° C]**	**ΔTm [° C]**	**SEM [° C]**	**ΔTm [° C]**	**SEM [° C]**
WT	11.3	± 0.1	10.1	± 0.1	9.7	± 0.1
F163C	9.2	± 0.1	7.3	± 0.1	5.9	± 0.1
F163D	8.5	± 0.3	5.0	± 0.1	4.0	± 0.1
F163E	9.9	± 0.0	5.1	± 0.1	7.2	± 0.1
F163G	8.8	± 0.1	4.6	± 0.2	5.0	± 0.2
F163H	9.5	± 0.1	6.0	± 0.1	6.4	± 0.1
F163I	8.4	± 0.1	4.6	± 0.1	3.1	± 0.1
F163L	9.1	± 0.1	5.3	± 0.1	2.1	± 0.2
F163N	9.3	± 0.1	6.3	± 0.1	6.2	± 0.1
F163Q	9.5	± 0.0	5.7	± 0.1	5.6	± 0.1
F163S	9.5	± 0.1	6.6	± 0.1	5.4	± 0.1
F163V	8.9	± 0.1	6.0	± 0.2	4.2	± 0.1
F163W	10.0	± 0.0	6.7	± 0.1	7.7	± 0.1
F163Y	9.6	± 0.1	8.2	± 0.1	8.9	± 0.1
I199V	10.6	± 0.1	8.7	± 0.2	9.3	± 0.1
F163Y-I199V	9.9	± 0.0	8.0	± 0.1	8.9	± 0.1
F163L-I199M	7.7	± 0.0	2.2	± 0.1	0.9	± 0.0
F163H-I199M	8.0	± 0.1	3.7	± 0.1	3.0	± 0.1
Q195A	9.2	± 0.2	7.9	± 0.1	8.7	± 0.2
D198A	7.0	± 0.1	7.0	± 0.1	6.0	± 0.1
R110A	1.7	± 0.1	-2.4	± 0.2	-1.0	± 0.2
R282A	2.2	± 0.1	1.4	± 0.1	5.0	± 0.1
R311A	-4.8	± 0.2	-4.9	± 0.2	-3.1	± 0.1
R110A-R282A-R311A	-6.1	± 0.2	-4.5	± 0.1	-3.3	± 0.3
